# Toxicity assessment of chlorpyrifos-degrading fungal bio-composites and their environmental risks

**DOI:** 10.1038/s41598-018-20265-9

**Published:** 2018-02-01

**Authors:** Jie Liu, Xiaoying Zhang, Mengran Yang, Meiying Hu, Guohua Zhong

**Affiliations:** Key Laboratory of Integrated Pest Management of Crop in South China, Ministry of Agriculture, South China Agricultural University, Guangzhou, 510642 P.R. China

## Abstract

Bioremediation techniques coupling with functional microorganisms have emerged as the most promising approaches for *in-situ* elimination of pesticide residue. However, the environmental safety of bio-products based on microorganisms or engineered enzymes was rarely known. Here, we described the toxicity assessment of two previously fabricated fungal bio-composites which were used for the biodegradation of chlorpyrifos, to clarify their potential risks on the environment and non-target organisms. Firstly, the acute and chronic toxicity of prepared bio-composites were evaluated using mice and rabbits, indicating neither acute nor chronic effect was induced via short-term or continuous exposure. Then, the acute mortality on zebrafish was investigated, which implied the application of fungal bio-composites had no lethal risk on aquatic organisms. Meanwhile, the assessment on soil organic matters suggested that no threat was posed to soil quality. Finally, by monitoring, the germination of cabbage was not affected by the exposure to two bio-products. Therefore, the application of fungal bio-composites for chlorpyrifos elimination cannot induce toxic risk to the environment and non-target organisms, which insured the safety of these engineered bio-products for realistic management of pesticide residue, and provided new insights for further development of bioremediation techniques based on functional microorganisms.

## Introduction

Organophosphate (OPs) are widely used in agriculture and household applications with a high share of global insecticide market. Due to their irreversible inactivation of cholinesterases (ChE) with consequent cholinergic hyper stimulation, OPs provide broad and effective control against pests for decades, guaranteeing world food supply and public health for a fast rising population. Chlorpyrifos (O, O-diethyl O-3,5,6-trichloro-2-pyridinyl phosphorothioate) is one of the largest consumed OPs that displays broad-spectrum insecticidal activity against important arthropod pests. In nature environment, the disappearance of chlorpyrifos includes evaporation, hydrolysis, oxidation, photolysis and microbial metabolism after direct soil application^1–3^. Owing to its low water solubility and high soil sorption, its off-site mobility (such as leaching and surface runoff) through and over soil profile is limited^3^, which may result in a high accumulation of chlorpyrifos residue for its intensive and excessive application in agricultural crops. In recent decades, growing evidence indicated that chronic exposure to chlorpyrifos can induce adverse effects on non-target organisms, which potentially threats the ecological balance^4–7^. Besides, the association between chlorpyrifos exposure and multiple clinical syndromes has attracted extensive concern since acute exposure to high chlorpyrifos levels leads to cholinergic crisis in human and animals including peripheral signs, central nervous system effects, and eventually convulsions and death^8–11^. In order to eliminate the pressure and potential threat of chlorpyrifos posing on ecosystem and human health, therefore, how to effectively reduce environmental chlorpyrifos residue has become a subject of intensive research.

Based on its physicochemical properties, multiple strategies via physical, chemical and/or biological treatments were broadly investigated so far, attempting to find efficient solutions for chlorpyrifos removal in realistic matrices^12–14^. Taking the advantages of plant/microbe metabolism, biological remediation has been extensively studied as abundant, permanent, safe, viable and non-invasive alternatives for pollutant elimination^15,16^. Among these, bioremediation by microorganisms possessing unique capacity of utilizing synthetic chemicals as their sole carbon and energy source has emerged as the most advantageous method for cleaning-up contaminants^17^. In order to manipulate their excellent enzymatic activity in realistic bioremediation, genetic engineering and molecular biological approaches have been employed to enhance their potential use, offering more effective and controllable methods for the bioremediation process^18–21^. However, various concerns and difficulties appear to impede these powerful techniques into realistic application, one of which is whether the introduction of non-indigenous microbes or engineered enzymes is safe to local ecosystem including mammals, plants, aquatic and soil organisms. Therefore, toxicity evaluation of proposed bioremediation skills is crucially necessary to understand the potential risks for the environment and non-target organisms prior to practical use, which can finally serve as the safe guide for the application of new bioremediation techniques.

Previously, we have fabricated two bio-composites composed of lyophilized fungi *Cladosporium cladosporioides* Hu-01 or its engineered enzyme to degrade chlorpyrifos with high efficiency, facilitating pesticide-decomposing microorganisms in practical application^22,23^. However, their toxic effects and environmental risks was rarely known. In this study, the toxicity of lyophilized fungi (LF) and purified enzyme (PE) bio-composites were assessed using mammals, aquatic organism, soil quality and plants, aiming to determine their acute and chronic toxicity upon non-target organisms as well as verifying the environmental safety of prepared bio-composites for future application.

## Results and Discussion

### Acute toxicity on mammals

In general, the acute toxicity of an unknown chemical or composite towards mammals were investigated via oral, ocular and dermal tests, in which mice and rabbits are commonly employed. Here, the mice were grouped by gender and given LF and PE once at 5 g kg^−1^ by gavage, respectively. Within 14 d, no dead or poisoned rat was found in treated groups, suggesting that the two fabricated chlorpyrifos-degrading bio-composites had no acute oral toxicity on rats (LD_50_ > 5 g kg^−1^). In order to determine the potential impacts of bio-composites on mice, more detailed indexes were investigated in terms of weight, daily food and water intake (in Fig. S1). Generally, slight fluctuations on three parameters were observed in both genders during experimental periods but no significant differences were obtained.

**Figure 1:**
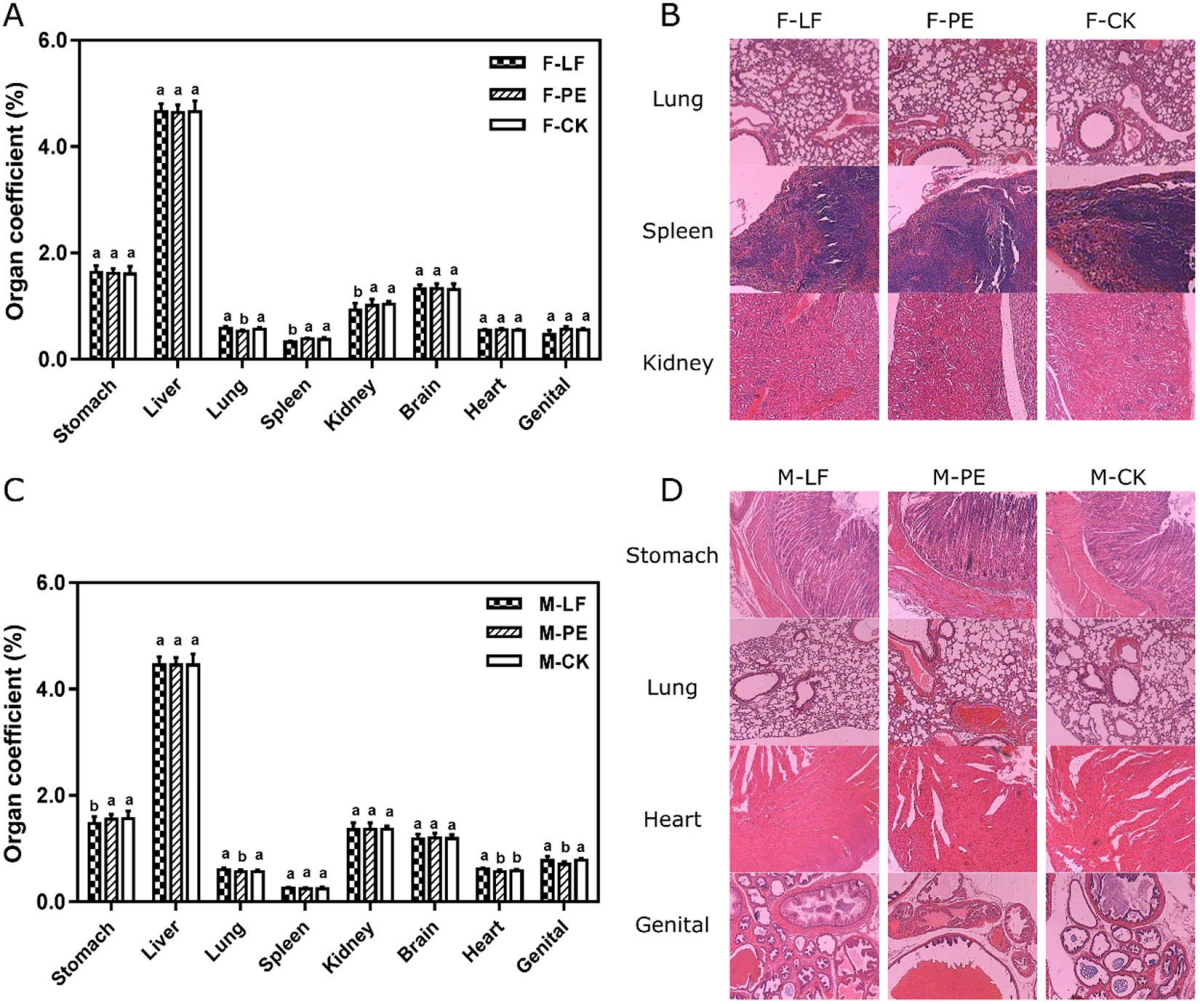
Oral toxic effects of LF and PE bio-composites on mouse organs. (**A**) Effects on organs of female mice and (**B**) paraffin sections of affected organs. (**C**) Effects on organs of male mice and (**D**) paraffin sections of affected organs.

The potential influences of LF and PE bio-composites on mouse organs were investigated after oral intake. Figure 1 illustrates the organ coefficients and relative paraffin slices after 14-day treatments in both genders. For the female mice, each organ coefficient displayed similar value as control group except those of lung, spleen and kidney (Fig. 1A). Specifically, the organ index of lung from PE-treated female was lower than the control while the coefficients of spleen and kidney were lower in LF treatments, but no significant pathological damage (such as bleeding, edema, etc) was found in related paraffin sections (Fig. 1B). As for the male groups, the organ coefficients of stomach, lung, heart and testicle were slightly different from the control (Fig. 1C). According to the micro-observation, no abnormal signs were exhibited in paraffin slices, implying no organic damage was caused for treated male mice (Fig. 1D). In common, the oral intake of LF and PE bio-composites indicated no acute lethal toxicity or organic damage in mice for both genders.

In addition, the possibility of two fungal bio-composites to induce acute ocular and dermal irritation were conducted using New Zealand rabbits. The stimulation caused by direct contact is manifested as inflammation. According to the symptoms (including conjunctiva congestion, iris hyperemia and corneal opacity) caused by short contact with strong stimulants, the level of inflammation could be classified to determine the eye irritation potential of applied stimulants^24,25^. In this case, the results in Table 1 indicated that the prepared LF and PE bio-composites induced mild conjunctiva congestion or discharge in rabbit eyes in 10 min, but these inflammation symptoms were reversible that recovered to normal status within 2 h. No iris hyperemia or corneal opacity was observed during experimental period (Fig. S2). Furthermore, New Zealand rabbits were used to determine the potential of two bio-composites causing acute dermal irritation. No erythema, eschar, edema or corrosion was observed 1 and 4 h after exposure to the test substances (Fig. S3). Thus, the direct contact towards two bio-composites scarcely caused ocular or dermal irritation in rabbits, which could be considered as safe formulations to mammals in short-time exposure.

**Table 1:** The ocular tolerability towards two bio-composites in rabbit eyes (n = 4).

Formulation	Time	Conjunctiva			Iris hyperemia	Corneal opacity
		Congestion	Swelling	Discharge		
Control	10 min	0	0	0	0	0
	2 h	0	0	0	0	0
LF	10 min	0	0	1	0	0
	2 h	0	0	0	0	0
PE	10 min	1	0	1	0	0
	2 h	0	0	0	0	0

### Accumulative and genetic effects on mice

**Figure 2:**
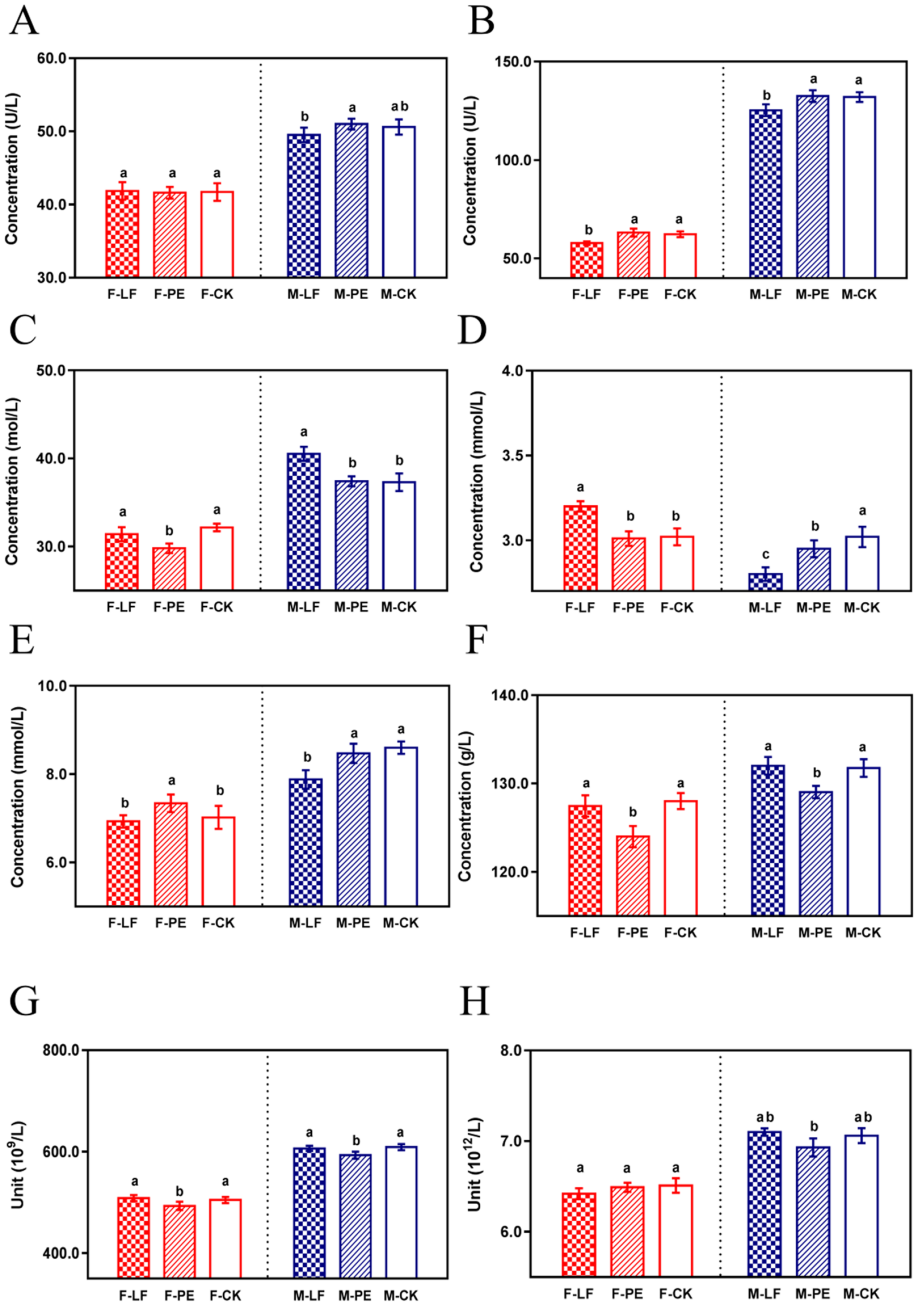
Assessment of mouse serum biochemistry and physiology after continuous oral intake of LF and PE bio-composites for 20 d. The levels of (**A**) ALT, (**B**) AST, (**C**) CRE, (**D**) GLU, (**E**) BUN, (**F**) HGB, (**G**) PLT and (**H**) RBC.

Due to the persistence of chlorpyrifos, the strategies for decontamination should aim at long-term performance, resulting in continuous exposure to the prepared fungal bio-composites. In order to clarify the accumulative effects of two bio-composites on mammals, the chronic mortality and serum biochemistry and physiology were investigated using Wistar mice. After 20 d, no dead or apparently poisoned mouse was found, suggesting the long-term contact to these two fungal composites barely induced lethal impact on mice. Blood is responsible for the delivery of necessary substances (such as nutrients and oxygen) and metabolic waste, the components of which can be directly or indirectly affected by the change of body tissues or organs. Figure 2 illustrates the results of serum biochemical and physiological parameters. Briefly, given the gender difference, the average alanine transaminase (ALT) levels in female mice (≈41.7 U L^−1^) were remarkably lower than that in male groups (≈51.6 U L^−1^), both of which were acceptable as the normal range of ALT (23.6 to 63.9 U L^−1^) (Fig. 2A). Similarly, the large gap between two genders revealed aspartate transaminase (AST) and creatinine (CRE) tests, but all locating in the normal ranges (53.0 to 153.3 U L^−1^ for AST and 21.6–51.0 M L^−1^ for CRE) (Fig. 2B,C). The glucose (GLU) levels (normally at 2.70~3.36 mM L^−1^) in control groups were determined the same (3.02 mM L^−1^) but the levels of treatments showed slight changes (Fig. 2D). In urea nitrogen (BUN, normally at 5.64~9.36 mM L^−1^) tests, PE-treated females demonstrated higher level (7.34 mM L^−1^) (Fig. 2E). In hemoglobin (HGB, normally at 113.23~146.6 g L^−1^) tests, mice treated with PE exhibited lower levels than LF-treated and control mice (Fig. 2F). Similarly, the concentrations of platelet (PLT, normally at 466.24~983.06 × 10^9^ L^−1^) and red blood cells (RBC, normally at 4.94~8.70 × 10^12^ L^−1^) in PE-fed mice were less than those in LF treatment and control (Fig. 2G,H). As for albumin (ALB), hematocrit (HcT), white blood cells (WBC), and lymphocyte (LY), no significant difference was determined (Fig. S4). Repeated and continuous contact to xenobiotics compounds below their sub-lethal concentration (LC_50_) values may cause accumulative toxicity since the chronic exposure may gradually exceed the threshold value to induce apparent poisoning and damage^26–28^. The blood tests showed no obvious abnormity since all values obtained were acceptable within the normal range, proving the chronic intake of two fungal bio-composites had negligible impact on mice.

**Figure 3:**
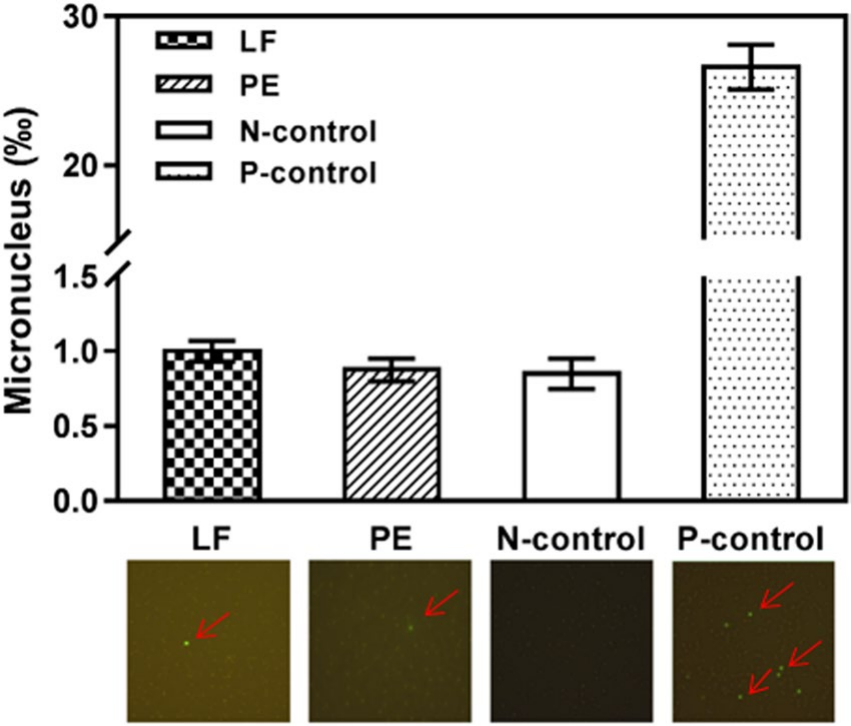
The micronucleus effects on mice bone marrow cells via chronic exposure to two fungal bio-composites. The micronucleus rates (top) and related micro-observation images (below).

Furthermore, the micronucleus test (MNT) was conducted using Wistar mice to determine the mutagenicity potential on mammal’s genetic and carcinogenic aspects^29,30^. Figure 3 illustrates the mice bone marrow micronucleus after gavage treatment. The micronucleus rate in positive control (cyclophosphamide) was determined at 26.6‰ with intensive spots under microscopy observation. In bio-composite treatments, the micronucleus rates were remarkably lower than positive control, implying that chronic contact towards fungal composites held less chance to cause genetic or carcinogenic influence. In summary, reliable evidences clarified that the chronic and continuous exposure of two fungal bio-composites had no accumulative toxicity or genetic effects.

### Effects on aquatic organism

Aquatic ecosystem is an important component in agriculture environment. In order to determine the aquatic safety of proposed bioremediation methods, zebrafish (*Danio rerio*) was employed to investigate the acute toxicity of LF and PE bio-composites after 24 and 48 h of exposure, respectively. Table 2 indicates that LC_50_ values of LF and PE bio-composites to zebrafish were determined to be 324.1 and 223.7 mg L^−1^ for 24 h while 204.6 and 170.8 mg L^−1^ for 48 h, respectively. The potential impact of two composites were intensified with increasing contact, but the sub-lethal dosages were high, which were classified as very low toxic to zebrafish (LC_50_ > 100 mg L^−1^)^31^. Therefore, the results indicated that these two fungal products were safe in aquatic ecosystem.

**Table 2:** The acute toxicity (48 h) of two chlorpyrifos-degrading bio-composites on zebrafish (n = 10).

Time (h)	Treatment	LC_50_ (mg L^−1^)	Regression equation	*R* ^2^	95% confidence limits (mg L^−1^)
24	LF	324.1	y = −6.534 + 2.603*x*	0.974	282.1~384.5
	PE	223.7	y = −6.140 + 2.613*x*	0.935	198.9~255.2
48	LF	204.6	y = −6.382 + 2.762*x*	0.972	139.7~360.6
	PE	170.8	y = −7.388 + 3.309*x*	0.960	121.1~262.7

### Effects on soil quality

**Figure 4:**
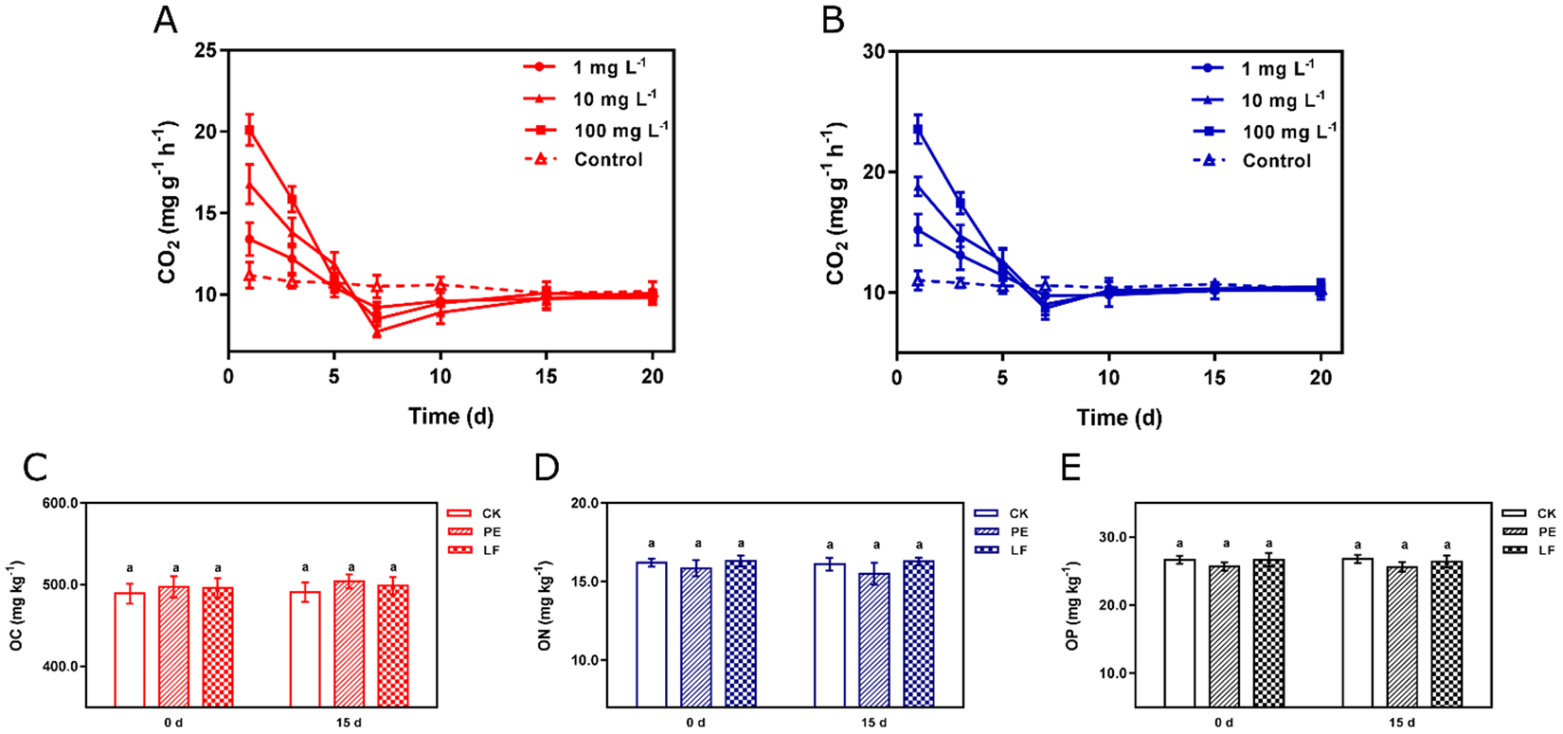
Effects of two fungal bio-composites on soil respiration and microbial biomass via chronic exposure. The effects on soil respiration caused by (**A**) LF and (**B**) PE bio-composites. The contents of (**C**) OC, (**D**) ON and (**E**) OP in soil.

Due to the metabolism of soil microbes in response to potential threats, basal soil respiration (BSR) and soil biomass are commonly used as “bio-markers” to assess soil quality under certain circumstances^32–34^. As shown in Fig. 4A,B, the BSR process was shortly affected that both composites had a short-term inhibitory effect on soil respiration rate within 7 d, which may be the reaction of soil microbes towards external stress. After 7-day exposure, the inhibition levels in all treatments gradually decreased and turned into stimulation of soil respiration, becoming normal and stable after 10 d with no remarkable difference to control group. Additionally, the levels of microbial biomass carbon, nitrogen and phosphorous were evaluated throughout 15 days. In Fig. 4C–E, the contents of organic carbon (OC), nitrogen (OC) and phosphorous (OP) remained the same after 15 d, indicating that the introduction of two bio-composites barely impacted the biomass elements in soil. Overall, these evidences showed that long-term exposure to two chlorpyrifos-degrading formulations could neither affect soil respiration nor alter the composition of biomass elements, which posed no harmful threat to soil quality.

### Potential effects on vegetables

One of the main applications of chlorpyrifos is to control pests on vegetable crops, which attracts raising concerns about the possibility of residue accumulation in farming field as well as the potential influence on vegetable growth. When employing bio-techniques to eliminate chlorpyrifos residue, therefore, it is important to understand the potential impact of applied fungus-based LF and PE bio-composites on vegetables. Here, the effects of two bio-composites on the germination and disease incidence (DI) of cabbage were investigated by using various dosages. As shown in Table 3, the germination rate of cabbage reached above 95% after 7 d of seeding in a dosage-related manner. From a practical point of view, the inhibition effect could be considered as negligible since the anti-germination rate was less than 5% in all dosage treatments. In addition, the DI of downy mildew (*Brassica rapa* ssp. *pekinensis*) was monitored in Chinese cabbage after 7 and 21 days, respectively (Table 3). The DI results indicated that approximately 16% of cabbage appeared disease signs that planted where pretreated with 10 mg L^−1^ of LF or PE bio-composites after 7 days, but showing no difference to control group. After 21 days of investigation, the DI of all bio-composite treatments remained relatively the same with a slight increase (DI < 18.5%), indicating the long-term exposure to two prepared bio-composites may not induce the rise of cabbage diseases. Meanwhile, the DI levels had no significant difference to control when treated with LF or PE at 10 mg L^−1^. Therefore, the present results confirmed that the use of both fungal composites in farming land was safe to the growth of vegetables.

**Table 3:** The seed germination and DI of downy mildew in cabbage under exposure of LF and PE bio-composites.

Treatment	Dosage (mg L^−1^)	Germination (%) (7 d)	DI (%)	
			7 d	21 d
LF	10	97.02 ± 0.60^a^	15.74 ± 0.45^d^	17.01 ± 0.07^cd^
	50	96.23 ± 0.54^b^	17.63 ± 0.23^a^	18.13 ± 0.25^b^
	100	95.91 ± 0.23^c^	17.01 ± 0.38^b^	17.83 ± 0.41^c^
PE	10	96.95 ± 0.36^a^	16.03 ± 0.42^cd^	16.94 ± 0.56^d^
	50	96.17 ± 0.43^b^	16.42 ± 0.31^c^	17.06 ± 0.93^cd^
	100	95.22 ± 0.51^c^	18.01 ± 0.28^a^	18.15 ± 0.51^a^
Control		97.14 ± 0.45^a^	15.82 ± 0.35^d^	16.81 ± 0.33^d^

Note: The data presented are the means with standard deviations of three separate experiments. Different letters indicate significant differences among dosages (*p* < 0.05, Duncan’s test).

## Conclusions

Here, we described the acute and chronic toxic effects of two fungus-based bio-composites used for the degradation of chlorpyrifos residue. Firstly, the acute oral, ocular and dermal impacts were investigated using mice and rabbits, showing no acute toxicity was induced through short-term contact on mammals. Secondly, mice were employed to evaluate the chronic effects on mammals by analyzing the accumulative and genetic toxicity, which evidenced that continuous exposure to the two bio-composites could hardly cause abnormal signs in mammals’ physiological and genetic status. Thirdly, we investigated the acute mortality on zebrafish, ensuring the application of bio-composites had no influence on aquatic organisms. Meanwhile, the quality assessment in soil showed no significant effects on soil microbial communities. Finally, Chinese cabbage was used to clarify that two bio-composites were unlikely to inhibit germination or cause plant diseases. In summary, the application of fabricated fungal LF and PE bio-composites scarcely induced toxic or adverse risk to environment and non-target organisms, providing new insights for the further development of bioremediation techniques based on functional microorganisms.

## Materials and Methods

### Chemicals and reagents

All chemicals were purchased from Sigma Aldrich (St. Louis, USA). The LF and PE bio-composites were prepared according previous studies^22,23^ and kept in 4 °C prior to use. Briefly, LF was a wettable powder formulation consisted of lyophilized fungi and other additive agents, which degraded 91% of residual chlorpyrifos on cabbage after 7 d of spray treatment^22^. As for PE bio-composite, which was composed of purified chlorpyrifos-degrading enzymes and other agents, could rapidly degrade chlorpyrifos in contaminated soil and pre-harvest cabbage with long shelf-life (5 months under 50 °C)^23^.

### Acute oral toxicity assessment on mice

Acute oral toxicity was assessed by using Kuming (KM) mice at average weight of 18~22 g which were inbred and provided by Center of Experimental Animals, Southern Medical University, Guangzhou China (License No. SCXK2006–0015). Following experimental protocol was approved by the Ethics Committee of Animal Experiment Center, South China Agricultural University (SCAU), which was carried out in accordance with the Regulations of Chinese Experimental Animals (2004) and Standards of Laboratory Animals in Guangdong Province (2010). After an adaptation period of one week, the animals were housed in wire-bottomed cages in a room under standard conditions at 25 ± 1 °C. Since there was no record about the toxic level of applied bio-composites, it was suggested to evaluate the maximum tolerated dose (MTD) which is defined as the maximum dose that cannot induce death of animal in toxicity test. If death or apparent poisoning signs occurred when MTD below 5 g kg^−1^, the Horn method was employed to determine the median lethal dose (LD_50_) of applied drug. Otherwise, the oral toxicity was negligible (MTD > 5 g kg^−1^). In this study, mice were divided into two genders and five in the same gender were fed together as a group. LF and PE bio-composites at 5 g kg^−1^ were once given to mice in both genders by gavage, respectively. The food and water was supplied routinely after 2 hours. Three replications were set for each treatment and the control group was fed the same volume of water without bio-composite. The survival, weight, food and water intake were monitored every 24 h for 14 days. In addition, the potential oral toxicity was investigated on mouse organs. After 14-day of feeding, the mice were killed by cervical dislocation and their organs were weighed individually including stomach, liver, lung, spleen, kidney, brain, heart and genital. Based on the weight of organs, the organ coefficients were calculated according to following equation (Eq. 1).

1 $${\rm{Organ}}\,{\rm{coefficient}}\,( \% )=\frac{{m}_{i}\,}{{m}_{o}}\times 100 \% $$

where *m*_*i*_ was the weight of organ (g) and *m*_o_ presented the weight of whole body (g). Meanwhile, those organs were observed for pathologic features and then fixed by paraffin embedding, which was eventually sliced and dyed (by haematoxylin and eosin) for micro-observation via optical microscope (ZEIZZ Axioskop2 plus, Hexmug, USA).

### Acute ocular and dermal toxicity on rabbits

Xenobiotic exposes to animals via multiple ways, such as oral intake and ocular/dermal contact. The latter may induce irritation and even corrosion in animal eyes and skin^35^. New Zealand rabbits (≈2 kg) were employed to evaluate the acute ocular and dermal toxicity of fabricated LF and PE composites previously dissolved in 0.9% NaCl at 1% and 5%, respectively. Those rabbits were inbred and purchased from Center of Experimental Animals, Southern Medical University, China (License No. 2007A001). Following experimental protocol was approved by the Ethics Committee of Animal Experiment Center, SCAU, which was performed in accordance with the Regulations of Chinese Experimental Animals (2004) and Standards of Laboratory Animals in Guangdong Province (2010). After an adaptation period of one week, the animals were housed under standard conditions at 25 ± 1 °C. 8 rabbits with normal eyes were chosen and divided into 4 groups. Each 0.1 mL of dissolved bio-composite was instilled into the conjunctival sac of right eyes while the left eyes were given 0.1 mL of 0.9% NaCl as control. Rabbit eyes was forced to close for 10 seconds. The status of conjunctiva, iris and cornea was observed at the intervals of 10 min, 1, 2, 4, 12, 24 and 48 h after giving stimulant. According to clinical signs (conjunctiva congestion, swelling, discharge, iris hyperemia and corneal opacity), the inflammation levels were graded from 0 to 4^24^. All measurements were conducted under the same environmental conditions by the same operator blind to treatment.

The potential of two formulations to cause dermal irritation was evaluated using New Zealand rabbits. Rabbit back were divided into left and right sections, and shaved at approximately 3 × 3 cm^2^. The dermal tests were conducted after shaving for 24 h with no swelling or redness signs. 0.5 mL of 1%/5% LF/PE composite was evenly applied on the skin of left section while the right side was applied with 0.5 mL of saline only, covered by a gauze patch and non-irritant tape. Each treatment contained 2 rabbits and triplicated. The patch was removed after 4 h and the residual substance was cleaned gently by saline. Irritation symptoms (erythema, eschar and edema) were evaluated and graded at 0, 4, 24 and 48 h after removing the patches as described by Betsabee *et al*.^25^.

### Accumulative and genetic effects on mice

The potential of two bio-composites for causing accumulative toxicity were investigated on Wistar mice which were inbred and purchased by Center of Experimental Animals, Southern Medical University, China (License SCXK2006B023). Following experimental protocol was approved by the Ethics Committee of Animal Experiment Center, SCAU, which was carried out in accordance with the Regulations of Chinese Experimental Animals (2004) and Standards of Laboratory Animals in Guangdong Province (2010). After an adaptation period of 1 week, the animals were housed in wire-bottomed cages in a room under standard conditions at 25 ± 1 °C. Since the MTD was tested above 5 g kg^−1^, LF and PE formulations were given to mice by daily gavage at 5 g kg^−1^ for 20 days while the control group was fed normally without fungal composites. Every treatment was conducted within 10 adult mice having both gender evenly and triplicated. The survival of mice and clinic symptoms were monitored every 24 h. After 20 day of feeding, 6 mL blood of each rat was drawn from the abdominal aorta, which was anaesthetized with barbiturates, i.e., 30 mg kg^−1^ body weight of sodium pentobarbital. Serum parameters including WBC, RBC, HGB, PLT, LY, HcT, ALT, AST, ALB, GLU, BUN and CRE, were analyzed by a Glamour Diatron LQ 06-Abacus junior VET analyzer (Diatron, USA) and an Automatic biochemical analyzer 7060 C (Hitachi, Japan) using diagnostic kits.

Meanwhile, MNT analysis was conducted among 60 Wistar mice with even genders at average weight of 25~30 g. Mice were divided randomly into following groups (10 animals per each): 1, Treatment A that once given with 5 g kg^−1^ of LF formulation by oral gavage; 2, Treatment B that once given with 5 g kg^−1^ of PE formulation by oral gavage; 3, Control N that given with volume saline as negative comparison, and 4, Control P that treated 60 mg kg^−1^ BW^−1^ of cyclophosphamide by intraperitoneal injection as positive control. Afterwards, all animals were fed routinely. The same treatments were repeated after 24 h. In the interval of 6 h after the second application, the mice were killed by cervical dislocation and the bone marrow cells were extracted from thigh bones by aspiration, placed in 1% sodium citrate and centrifuged for 5 min at 1000 rpm. As Chinde and Grover described previously, the cell pellet was re-suspended, dyed and finally prepared as slice for micro-observation^36^. For each rat, three slides were prepared. Randomly 1000 polychromatic erythrocytes (PCEs) per animal from each slide and the frequency of micronucleated PCEs (MN-PCEs) were noted. The micronucleus rate (‰) was analyzed as equation below (Eq. 2).

2 $$\mathrm{Micronucleus}\,\mathrm{rate}\,({\rm{\textperthousand }})=\frac{{N}_{MN-PCEs}}{{N}_{PCEs}}\times 1000$$

### Acute toxicity on zebrafish

Zebrafish (*Danio rerio*) was selected as a model for the assessment of acute toxicity on aquatic organisms^31^, which were inbred and provided by Animal Experiment Center, SCAU. The related protocol was approved by the Ethics Committee of Animal Experiment Center, SCAU, which was carried out in accordance with “Laboratory Fish Part 6: Environmental and Housing Facilities (DB11/T 1053.6–2013)” and “Test guidelines on environmental safety assessment for chemical pesticides Part 12: Fish acute toxicity test (GB/T 31270–2014)”. Healthy adult fish were selected and acclimatized in glass tanks at ambient temperature (27 ± 1 °C) with 14-h light/10-h dark cycles for one week, and stopped feeding 1 day before test. When conducting the test, every 10 fish were transferred into a 10-L glass tank equipped with cover as a group. LF and PE bio-composites were applied at gradient 0, 10, 20, 50, 100, 200, 400 and 1000 mg L^−1^, respectively. Each concentration set 3 repeats without feeding during the test period. Potassium dichromate was used as positive control. The survival of fish and possible poisoning signs in each group was monitored and noted after 24 and 48 h, which were used to analyze the concentration that caused half of lethality termed as LC_50_.

### Soil respiration and microbial biomass

Soil samples were taken from top 20 cm soil at SCAU research farm, Guangzhou China without agricultural chemical application. The soil was composed of 18.2% sand, 73.1% silt and 8.7% clay. Basal soil respiration (BSR) of samples were measured by trapping the evolved CO_2_ in NaOH^37^. Every 50 g of sieved soil (4 mm mesh) was prepared in 1000-mL wide-necked bottle and soil moisture was adjusted to 60% maximum water holding capacity. 1, 10 and 100 mg kg^−1^ of LF and PE bio-composites dissolved in 1% glucose solution was added into bottles, respectively to saturate the catabolic enzymes of the microorganisms^38^. Each concentration consisted three repeats with control group that given the same volume of deionized water. A tube containing 30 mL of 0.1 M NaOH was placed into each bottle to trap the evolved CO_2_. All the bottles were sealed with vaseline and incubated in darkness at 25 °C. The content of CO_2_ was titrated after 1, 3, 5, 7, 10, 15 and 20 d with 0.1 M HCL using phenolphthalein as indicator. According to previous methods^39,40^, microbial biomass of the soil samples was determined by measurement of ninhydrin-reactive N following fumigation with liquid chloroform and extraction with potassium chloride. Briefly, three subsamples of soil (5 g) from the different treatments were weighed into Erlenmeyer flasks with 1 mL chloroform and three same mass of control soil were prepared as well. All flasks were sealed and evacuated in a vacuum desiccator for 10 seconds. The whole set was incubated in dark for 15 d at 25 °C. Then, 0.5 M potassium chloride (*w*/*v*, 1/4) was added to each flask, shaken for 1 h and then centrifuged. The detailed determination of microbial biomass carbon, nitrogen and phosphorus was conducted as described by Dutta *et al*.^37^, Eisenhauer *et al*.^38^ and Singh *et al*.^39^.

### Germination and disease incidence of cabbage

Every 30 cabbage seeds were placed on a moisturized filter paper in a 9-cm Petri dish. 2 mL of 10, 50 and 100 mg L^−1^ of LF or PE bio-composite was used to moisturize the filter paper, respectively. Every treatment repeated three times with control group that given 2 mL of deionized water. After 7 d, the germination rate in each group was noted, which expressed as the percentage of germinated seeds to the total seeds.

Every five seedlings which was not treated with fungal composites were transferred and planted into a pot. When the cabbage was grown to 3–5 leaves, 10, 50 and 100 mg L^−1^ of LF or PE bio-composite was sprayed to cabbage leaves and incubated at 25 °C. The DI of downy mildew in cabbage was monitored based on the area of yellowing, disease spots and rot on infected leaves after 7 and 21 days of first spray, every 5 leaves (from top to bottom) from each plant were randomly selected for DI investigation. The following equations were employed to calculate the DI on cabbage.

3 $$\mathrm{DI}\,( \% )=\frac{{\sum }^{}({P}_{k}\times k)}{{N}_{P}\times 9}\times 100 \% $$

where *P*_*k*_ presents the number of infected leaves, *k* is the value of infection level and *N*_*p*_ is the number of total leaves. The infection level was classified as following: 0, no disease sign; 1, one third of infection area; 2, two third of infection area, and 3, total area of infection.

### Data analysis

Standard deviations were determined using Statistic Analysis System (SAS). The significance (*p* < 0.05) of differences was treated statistically by one-way analysis of variance (ANOVA) and evaluated by post hoc comparison of means using Duncan’s test by Statistical Product and Service Solutions (SPSS) software.

### Data availability statement

All the authors have claimed that the data in present manuscript were available. The requests for materials should be addressed to G.Z.

## Electronic supplementary material

Supplementary information
