# Supplementary material for: Toxicity assessment of chlorpyrifos-degrading fungal bio-composites and their environmental risks
Source: Sci Rep. 2018 Feb 1;8:2152. doi: 10.1038/s41598-018-20265-9 (PMC5794795; doi:10.1038/s41598-018-20265-9)
Supplement: Supplementary file 1 — Supplementary information [file 41598_2018_20265_MOESM1_ESM.pdf]

Toxicity assessment of chlorpyrifos-degrading fungal bio-composites and their environmental risks

Jie Liu[1]*, Xiaoying Zhang*[1]*, Mengran Yang, Meiying Hu, Guohua Zhong**

Key Laboratory of Integrated Pest Management of Crop in South China, Ministry of Agriculture; Key Laboratory of Pesticide and Chemical Biology, Ministry of Education; South China Agricultural University, Guangzhou 510642, P. R. China

[1]These authors contributed equally to this work.

*Correspondence and requests for materials should be addressed to G.Z.

Email: guohuazhong@scau.edu.cn

Tel: +86 020 8528 0308

Supporting figures

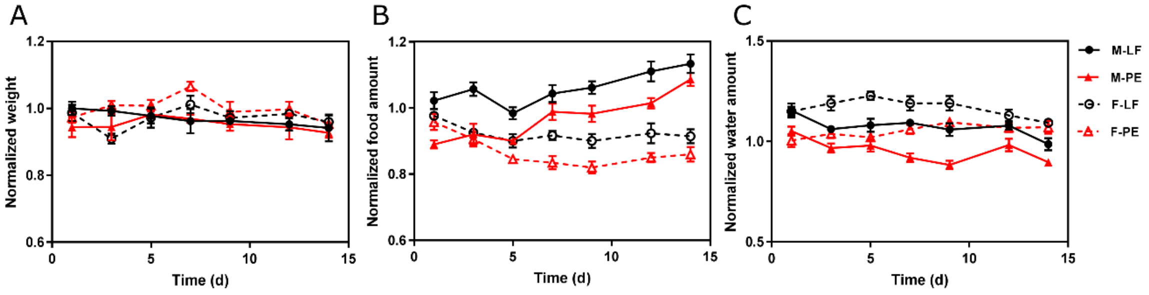

Fig. S1 Oral toxic effects of LF and PE bio-composites on mice during 14 days. The effects on (A) weight, (B) food intake amount and (C) water intake amount.

From **Fig. S1A**, the weight of male rats fed with two bio-composites remained steady compared to control group while the weight of female mice displayed slight fluctuations within observation period, but no significant difference (p < 0.05) was obtained in both genders. In addition, the amounts of daily food intake were recorded in **Fig. S1B**, which showed gradual increase within first 5 days and then became steady in female groups. Differently, the food amount of male rats increased by almost 110% after a slight fall in first 5 days. However, the food intake of mice treated with lyophilized fungal composite was relatively higher than those fed with enzymatic formulation, which was in common for both genders. Besides, the volume of daily water intake indicated a few fluctuations within two weeks in **Fig. S1C**. Generally, the male rats tended to drink more water compared to the females when being treated with the same bio-composite, whilst those once given the LF preparation exhibited higher requirement of water than those with PE treatment in both genders.

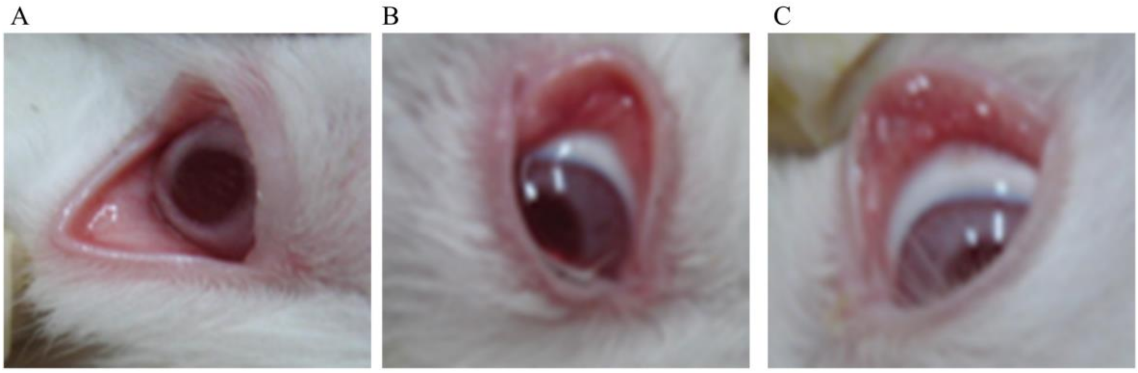

Fig. S2 Ocular effects of two bio-composites on rabbit eyes after 2 h. (A) Control, (B) LF-treated eye and (C) PE-treated eye.

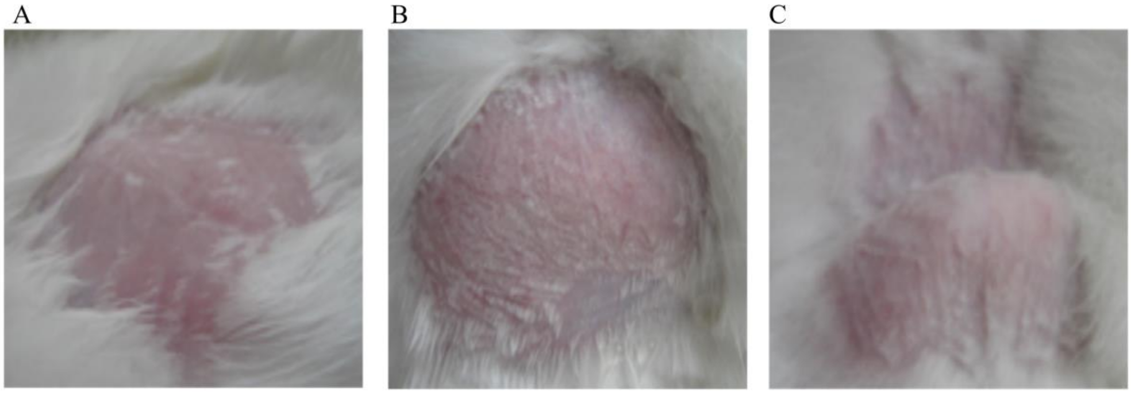

Fig. S3 Dermal effects of two bio-composites on rabbit skin after 4 h. (A) Control, (B) LF-treated eye and (C) PE-treated eye.

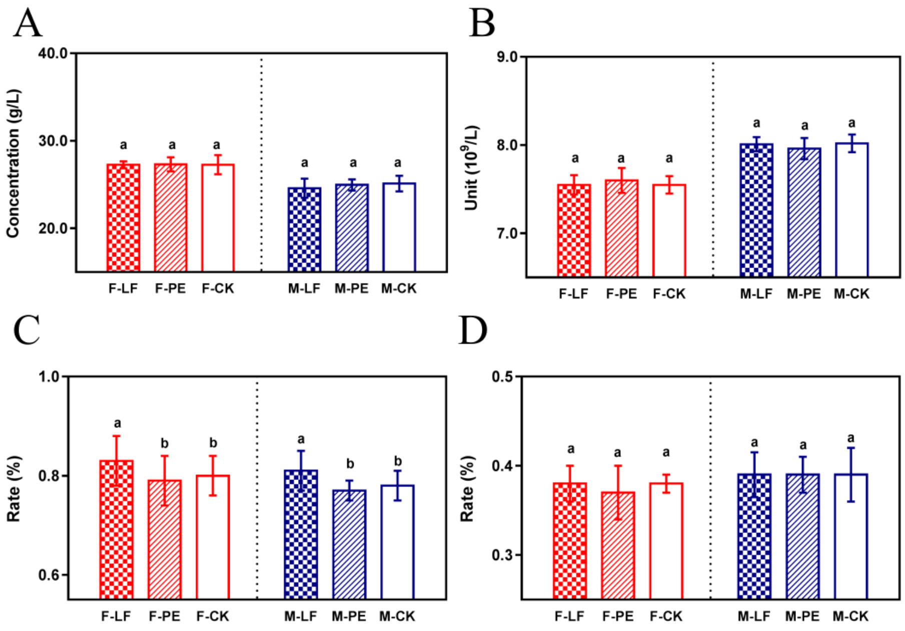

Fig. S4 Assessment of mouse serum biochemistry and physiology after continuous oral intake of LF and PE bio-composites for 20 d. The levels of (A) ALB, (B) WBC, (C) LY and (D) HcT.
